# Patient Benefit Following Bimodal CI-provision: Self-reported Abilities vs. Hearing Status

**DOI:** 10.3389/fneur.2018.00753

**Published:** 2018-09-10

**Authors:** Elisabeth Wallhäusser-Franke, Tobias Balkenhol, Svetlana Hetjens, Nicole Rotter, Jerome J. Servais

**Affiliations:** ^1^Department of Otorhinolaryngology Head and Neck Surgery, Medical Faculty Mannheim, University Hospital Mannheim, Heidelberg University, Mannheim, Germany; ^2^Institute of Medical Statistics and Biomathematics, Medical Faculty Mannheim, Heidelberg University, Mannheim, Germany

**Keywords:** cochlear implant, hearing aid, bimodal hearing, SSQ, speech audiometry, anxiety, depression, general self-efficaciousness prospective study

## Abstract

**Objectives:** Patient-reported outcomes gain importance for the assessment of auditory abilities in cochlear implant users and for the evaluation of auditory rehabilitation. Aims of the study were to explore the interrelation of self-reported improvements in auditory ability with improvements in speech comprehension and to identify factors other than audiological improvement that affect self-reported auditory ability.

**Study Design:** Explorative prospective analysis using a within-subjects repeated measures design.

**Setting:** Academic tertiary care center.

**Participants:** Twenty-seven adult participants with bilateral sensorineural hearing loss who received a HiRes 90K CI and continued use of a HA at the non-implanted ear (bimodal hearing).

**Intervention:** Cochlear implantation.

**Main Outcome Measures:** Self-reported auditory ability/disability assessed by the comparative version of the Speech, Spatial and Qualities of Hearing Scale (SSQ-B), and monosyllable as well as sentence comprehension in quiet and within speech modulated noise from different directions assessed pre- as well as 3 and 6 months post-implantation.

**Results**: Data of 17 individuals were analyzed. At the endpoint of the study, improvement of self-reported auditory ability was significant. Regarding audiometric measures, significant improvement was seen for CI-aided pure tone thresholds, for monaural CI-assisted and bimodal sentence comprehension in quiet and in speech-modulated noise that was presented from the same source or at the side of the HA-ear. Correlations between self-reported and audiometric improvements remained weak, with the exception of the improvement seen for monaural CI-aided sentence comprehension in quiet and self-perceived improvement of sound quality. Considerable correlations existed between self-reported improvements and current level of depression and anxiety, and with general self-efficaciousness. Regression analyses substantiated a positive influence of self-efficaciousness on self-reported improvement in speech comprehension and between the improvement of monaural CI-aided sentence comprehension in quiet and perceived sound quality as well as a negative influence of anxiety on self-reported improvement in spatial hearing. Self-reported improvements were significantly better in the subgroup with intensive as compared to regular rehabilitation.

**Conclusions:** Self-reported auditory ability/disability represents an important measure for the success of bimodal CI-provision. It is influenced by personal and mental health factors that may improve CI-rehabilitation results if addressed during rehabilitation.

## Introduction

Aim of provision with assistive hearing devices is to reinstate auditory function and to improve communication and participation for the individual patient. With technological progress, relaxation of criteria for cochlear implantation and the aim to achieve binaural hearing, bimodal provision, i.e., a cochlear implant (CI) in one ear and a hearing aid (HA) at the other ear, is used by a growing number of CI-patients. Overall, this combination of electrical and acoustical hearing has benefits, but this is not necessarily true for each user and all types of conditions (1–7). Given the relatively limited body of literature on bimodal hearing and because of progress in CI- and HA-technology, further research on the benefits of bimodal hearing is warranted.

Clinical routine outcomes consist of CI- and HA-aided speech comprehension in quiet and noise. In research studies, performance in audiometric tests shows substantial dependence on the type of speech material, on the nature of noise confounders, and on spatial distribution of speech and noise sources (8–10). Furthermore, during audiometric tests pronunciation is clear, spectro-temporal characteristics and location of the speaker as well as noise type are static and thus predictable, and predictability increases the performance of CI-patients. In contrast, in daily life auditory sources may move and rapid switches between sources are common. Thus, audiometric tests may not be representative for the benefit subjects perceive from assisted hearing in their daily life. Therefore the relation of performance in audiometry with self-perceived auditory abilities is of interest. Consequently, emphasis on patient-centered perspectives is increasing. This is even more important as subjectively perceived benefit is likely related to acceptance and consequently long-term regular use of the hearing devices as opposed to their refusal.

Impact of hearing impairment can be divided into 3 domains (11, 12). One is hearing status, the other is perceived ability or disability, and the third is perceived personal handicap. Whereas hearing status should be closely linked to audiometric performance, it is intuitive that the latter domains are likely influenced by a variety of non-auditory factors like character, expectations, and life circumstances. In addition, interaction with psychosocial health can be expected (11, 13–16). In this context questionnaires assessing self-reported auditory abilities are a valuable tool.

One such instrument is the Speech, Spatial, and Qualities of Hearing Questionnaire (SSQ: 12). This questionnaire focusses on functional auditory abilities in environmental situations. Auditory situations captured by the SSQ include single and multiple talker situations with or without background noise, localization of stationary and moving auditory targets as well as quality and clarity of sounds, including situations that are known to be challenging to the hearing impaired. In contrast to other questionnaires which address both disability and handicap [e.g., (13, 17)] the SSQ focusses exclusively on functional auditory abilities, avoiding confusion of ability/disability and handicap. The questionnaire has been widely used internationally and there exists a validated German translation (18). It was shown to be sensitive to binaural abilities (19) and to change of auditory performance in CI-patients (20). Moreover, a benefit version, the SSQ-B, has been developed for the assessment of self-perceived improvements following an intervention (21, 22). At present, few studies have examined both audiometric and self-reported outcomes assessed with the SSQ for bimodal CI-users (5–8, 23–30). These studies evidence large inter-individual variability as well as inconsistent connections between audiometry and self-report. Therefore, the extent of association between these measures and their relevance as clinical outcomes needs to be elaborated.

The following report presents the audiometric data of a study that was designed to evaluate transition from HA-assisted to bimodal hearing. Primary aim was to establish the association between self-reported improvements in auditory ability with improvement in hearing status assessed by speech audiometry during the first months of bimodal hearing in adult CI-users. Second aim was to identify factors other than audiological improvement that may affect self-reported improvement of auditory ability.

## Subjects and methods

### Procedure and inclusion

Before initiation of the study, the study protocol was approved by the Institutional Review Board of the Medical Faculty of Mannheim at Heidelberg University (approval no. 2014-527N-MA). Prior to inclusion, each subject provided written consent for participation in the study that was conducted in accordance with the Declaration of Helsinki. Participants were compensated for their time at test days T3 and T4.

Between 2014 and 2017, study participants were recruited from the patients at the CI Center of the University Medical Centre Mannheim (KCIM). Prospective participants were adults with acoustic auditory experience. Inclusion criteria comprised first-time unilateral CI provision, a HiRes 90K implant as chosen by the patient, continued HA use at the other ear, and aged between 18 and 90 years. All patients who fulfilled these criteria were approached for inclusion. Exclusion criteria were assessed during an initial interview (T1) and were insufficient knowledge of the German language and more than mild cognitive deficit, as assessed by the DemTect Test (31). The initial interview, study inclusion (T1), and pre-surgery examination (T2) took place at the same day, usually the day before surgery (Table 1).

**Table 1:** Participants' characteristics.

Gender: female/male N	13/4
Cohabitating N	13
Age M ± SD (range) in years	58.06 ± 15.00 (27–78)
Cochlear implant (CI) left/right N	9/8
Days between implantation and assessment	T2:2.65 ± 6.79 (1–29) T3:93.00 ± 17.07(75–145) T4:228.35 ± 74.74(170–427)
CI ear: years with hearing impairment	25.12 ± 17.98 (2–56)
HA ear: years with hearing impairment	21.75 ± 18.98 (1–56)
Pre-OP HA use on CI ear (excluding CROS) N	13
CI-ear: PTA4 (dB HL)	
Pre-implantation	96.43 ± 16.20
Post-implantation	44.17 ± 12.96
HA-ear: PTA4 (dB HL)	
Pre-implantation	67.33 ± 17.43
Post-implantation	60.47 ± 21.42
HADS-Anxiety at T2 and T4	T2: 5.36 ± 4.11; T4: 4.35 ± 2.91
HADS-Depression at T2 and T4	T2: 4.59 ± 4.29; T4: 3.88 ± 3.84
General Health Rating at T2 and T4	T2: 2.35 ± 0.93; T4: 2.58 ± 1.00
Relevant other health conditions N	9
Tinnitus N	14

Patients received a CI on their weaker ear while HA use was continued on the other ear. They left hospital on average 3 days post-surgery. Two to three weeks later, they participated in a week-long in-patient program with first fitting of the speech processor, several fitting sessions, and technical instruction on CI use. Post-implantation assessments T3 and T4 were scheduled for 3 and 6 months post-implantation respectively (Table 1). Between T3 and T4, about half of the participants took part in an in-patient program at a specialized CI-rehabilitation clinic (intensive rehabilitation), whereas the other half used regular out-patient CI-rehabilitation services (regular rehabilitation).

The same speech comprehension tests were performed at the assessments pre- (T2) and post-implantation (T3, T4), and the questionnaires outlined below were completed within the same test session. At all appointments, tinnitus characteristics, and burden were assessed via questionnaires. Change of tinnitus following CI-provision is subject of a separate publication (32).

### Subjective improvements of auditory performance

At T4, the benefit version of the Speech, Spatial, and Qualities of Hearing Scale [SSQ-B: 12, 21, 22] was used to assess subjectively perceived improvement of auditory communication in the bimodal listening condition (LC) as compared to pre-surgery HA-assisted hearing. This comparative version contains the same 49 questions as the SSQ and is divided into the same 3 subcategories, namely speech understanding (SSQ-B1), spatial hearing (SSQ-B2), and sound quality (SSQ-B3). Comprehending speech in a range of realistic conversational situations involving single and multiple talkers in quiet and noisy surroundings, including reverberation is addressed by 14 items (SSQ-B1), 17 items (SSQ-B2) cover directional and distance aspects of stationary and moving sound sources, and 18 items (SSQ-B3) examine the degree of sound quality or clarity through various types of sound, as well as separation of sounds and listening effort. In the SSQ-B, subjects are asked whether the situation has changed as compared to pre-CI hearing. Responses are indicated on a rating scale from −5 to +5. Positive scores indicate improvement, while negative scores indicate worsening. A score of 0 represents no change. For all questions there is an option to tick “not applicable”. For each scale, means were calculated from single item scores. In addition an overall mean score (SSQ-B_mean_) was calculated from the means of the three scales. Study participants filled out a paper-and-pencil version of the questionnaire by themselves along with the other questionnaires. They were invited to consult an investigator if in doubt.

### Audiometric improvements

All participants were implanted with a HiRes 90K implant and used the NAIDA Q70 speech processor. At T2 one (*N* = 4) or both ears (*N* = 13), and at T3 and T4 the non-implanted ear was aided by a HA. Devices were used with participants' typical daily settings during the course of testing. During audiometric testing participants were seated comfortably in a dimly lit double-walled sound attenuating booth (IAC Acoustics).

#### Pure tone audiometry

Aided thresholds at 0.5, 1, 2, and 4 kHz (mean: PTA4) were recorded separately for each ear in free sound field using standard audiometric procedures (Audiometer Auritec AT900). PTA4 was measured before CI-provision and after switch-on of the implant, typically 3–4 weeks following CI surgery. Before surgery, thresholds could not be determined in 9 of the 17 future CI ears and in 1 HA ear due to no response at some or all of the PTA4 frequencies. For the calculation of overall improvement in hearing, values for measurements that did not yield a result were set to 120 dB HL.

#### Speech comprehension

At T2 only the best-aided binaural LC was tested, whereas at T3 and T4, bimodal (CI and HA) and both monaural LCs (CI only, HA only) were assessed separately. During the monaural HA condition, the external part of the CI was removed, while for the monaural CI condition the HA was removed and the ear was plugged with an in-ear headphone (AKG K350, earplug from GSI Grason-Stadler) through which white noise at 65 dB was presented, calibrated with a Brüel and Kjær Type 2250 sound level meter with custom tube adaptor. The sequence of device and noise conditions and test lists was counterbalanced across participants and across test blocks. Testing for one device condition was completed before shifting to another condition.

In all tests, speech was presented from a nearfield studio monitor loudspeaker (M-AUDIO BX5) placed one meter in front of the participant (S0). Before each test session, sound pressure level was calibrated with OlSa calibration noise and a Brüel and Kjær Typ 2250 sound level meter. Lateral noise signals were presented via additional loudspeakers (M-AUDIO BX5) placed at ± 90° with a distance of 1 m to subjects ears. Speech comprehension in quiet and with competing noise was tested with a monosyllable test [Freiburger Monosyllable [FBE]; (33)] and a matrix sentence test [Oldenburg Sentence Test [OlSa], (34–36)]. Listeners verbally repeated the word (FBE) or each word in the sentence (OlSa) as understood, with the experimenter entering the total number of keywords correctly identified following each trial. No feedback was given, lists were not repeated within sessions.

During FBE tests, two lists of 20 words, spoken by a male talker were presented at 70 dB SPL for each LC. In the FBE, higher percentages represent better understanding. During OlSa tests, sentences were presented by a male talker. The 50% speech reception threshold (SRT) for comprehension in quiet and the signal to noise ratio (SNR) for 50% correct comprehension in noise were determined adaptively with lower values representing better results. For the speech in noise conditions, speech-shaped OlSa noise with the same frequency distribution as the OlSa sentences (34–36) was used, and presented simultaneously with a constant level of 60 dB from the same loudspeaker as the speech signal (S0N0), or from a loudspeaker facing the CI (S0NCI) or the HA ear (S0NHA), respectively.

OlSa sentences consist of five-word nonsense sentences with identical structure and with 10 possible words per position, yielding a high number of different sentences and low predictability of single words. Beginning with a speech level of 70 dB SPL, presentation level of sentences was decreased if more than three words were recognized correctly, while it was increased if fewer than two words were recognized and held constant in between. Twenty sentences were presented per condition. SRTs and SNRs were calculated as the average presentation level of the 10 final sentences. If curves did not show turning points, the SRT or SNR for that condition was determined with a second, different OlSa list.

### General health

General health was assessed at T2 and T4 by one question asking how the subject judged his or her general health condition. Possible answers were poor (0), moderate (1), ok (2), good (3), and very good (4).

Also at T2 and T4, the Hospital Anxiety and Depression Scale [HADS, (37)], a validated and widely used questionnaire that assesses symptoms of depression (HADS-D) and anxiety (HADS-A) in clinical samples was used to uncover potential problems in these areas. For both scales, higher scores represent a higher level of symptom and indicate poorer mental health.

In addition, perceived general self-efficaciousness was assessed with the 10-item GSE scale by Schwarzer and Jerusalem (38). General self-efficaciousness describes the belief of an individual in his or her capacity to deal effectively with challenging situations (38). The GSE scale exists in many languages, has been used in many studies, and was validated in a multicultural validation study (39). Response options are not true (1), hardly true (2), rather true (3), and exactly true (4) with the 10 items adding to a sum score between 10 and 40 higher scores represent better outcomes.

### Statistical analysis

Analyses were performed with SPSS24 (SPSS/IBM, Chicago, IL, USA). Group means (M) are presented together with their standard deviations (SD) in tables, and with their standard errors (SEM) in figures.

For PTA4, OlSa tests, and the HADS questionnaire, low numbers indicate favorable conditions, while higher numbers represent better outcomes in the FBE test, for global health, GSE, and in the SSQ-B. All audiometric improvements were calculated to be positive with higher numbers indicating larger improvements and negative numbers indicating worsening. When assessing the impact of CI-provision on normally distributed data, a paired *t*-test or one-way repeated measures ANOVA were used to determine significance. If one data set did not have a normal distribution, non-parametric Friedman tests with Wilcoxon matched-pairs signed rank tests were used instead. For main effects *p*-values below 0.05 were considered to be statistical significant, while *p*-values below 0.01 were considered as highly statistically significant. If significant main effects were present, *post-hoc* tests with Bonferroni correction, i.e., division of *p*-value by number of tests, were applied.

To further examine interrelations among improvement of self-reported auditory ability and audiological improvements, and relationships between perceived improvement and health factors, bivariate Pearson correlation analyses were conducted with the mean score (SSQ-B_mean_) of the three SSQ-B scales (Table 3). Pearson correlation coefficients *r* < 0.5 are considered as weak, coefficients between 0.5 and 0.8 are considered as moderate, and *r* > 0.8 are considered as strong.

Finally, linear regression analyses were performed separately for each of the SSQ-B1-3 scales with the factors that achieved a Pearson correlation coefficient *r* > 0.5 and a *p*-value *p* < 0.05 in the bivariate correlations with SSQ-B_mean_. These variables are depicted in bold in Table 3.

### Participant characteristics

Twenty-seven patients with hearing loss at both ears who planned to undergo unilateral implantation of a HiRes 90K CI were screened. One was excluded because of an exclusion criterion and 26 were included in the study. Two discontinued following sequential bilateral implantation, one decided that study participation after T2 was too much effort, two discontinued for reasons they did not disclose, and one was excluded because of an exclusion criterion that had not been disclosed before. Complete data sets were available for 20 participants, of whom data for 3 were excluded because of an incidence of sudden hearing loss (SHL) in the non-implanted ear associated with Meniere's disease, because of not using the HA at the non-implanted ear at T4, or because of substantial changes in loudness tuning of the HA between T3 and T4. Demographic information relating to etiology and onset or diagnosis of hearing loss, pre-implant acoustic amplification and the time between assessments and surgery for the remaining 17 participants is provided in Table 1.

#### History of hearing loss

Six participants reported hearing problems since early childhood, while 11 had post-lingual onset of profound hearing impairment. At inclusion in the study all participants could communicate verbally when using HA. Causes for hearing loss were unknown for most (*N* = 12), or were due to SHL (*N* = 3), Meniere's disease (*N* = 2), and Stickler Syndrome (*N* = 1). On average severe hearing impairment of the CI ear existed for 25 years, while duration of severe hearing impairment at the HA ear yielded an average of 22 years (Table 1).

## Results

Until the first formal appointment at the KCIM 4 weeks following surgery, participants' mean daily processor use was 11 h. At T4, 15 participants reported daily combined use of CI and HA for more than 8 h. CI and HA were always used together by 8, while 8 reported situations during which use of the HA was inconvenient. Most commonly this occurred during conversations in quiet. On a scale from 0 (no change) to +5 (more content) or −5 (less content) satisfaction with the CI was higher (2.63 ± 1.41) than with the HA (0.65 ± 2.12), or with the combination of both devices (1.82 ± 1.70). Life quality had improved for 11 and remained unchanged for the other 6 subjects.

### SSQ-B

**Figure 1:**
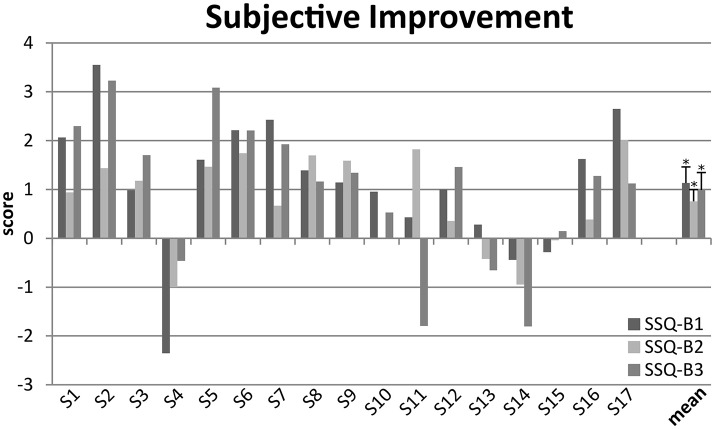
Individual and mean self-reported improvements assessed with the SSQ-B at T4 for speech comprehension (SSQ-B1), sound localization (SSQ-B2) and sound quality (SSQ-B3). Individual means (S1–S17), and group means together with their standard errors are shown for each of the SSQ-B1-3 scales. Group means evidence improvements that are significantly different from 0 (*p < 0.05).

At T4, study participants judged improvement of their hearing in everyday listening situations with the SSQ-B questionnaire. Missing responses because the situation was not applicable comprised 3.4% of total possible responses in SSQ-B1, 5.5% in SSQ-B2, and 1.6% in SSQ-B3. Subjective improvement due to bimodal hearing as compared to pre-surgery HA-assisted hearing was highest for speech comprehension (SSQ-B1: 1.13 ± 1.20), lowest for the localization of auditory objects (SSQ-B2: 0.76 ± 0.89), and intermediate for perceived sound quality (SSQ3-B: 0.99 ± 1.33). Results of Pearson correlation analysis evidenced significant interrelations among the 3 scales (*r* > 0.7; *p* ≤ 0.001). At group level, improvements in all SSQ-B scores were significantly different from zero (SSQ-B1: *t* = 3.388; *p* = 0.004; SSQ-B2: *t* = 3.062; *p* = 0.007; SSQ-B3: *t* = 2.634; *p* = 0.018). There were obvious individual differences, however. Whereas 11 subjects reported an improvement exceeding 1.0 in at least one SSQ-B scale; 2 participants (S4, S14) reported worsening in all scales exceeding 1.0 in at least one scale; results were mixed for one (S11), and changes were minor for 3 (S10, S13, S15; Figure 1).

### Pure tone audiometry

Improvement of PTA4 with CI use was highly significant for the CI ear (Table 1: *z* = 3.621; *p* < 0.001), while HA-aided PTA4 at the contralateral ear remained the same (Table 1: *t* = 0.995; *p* = 0.335). Asymmetry between the PTA4 of both ears expressed as group average did not decrease with 29.19 dB (± 24.95 dB, range from 0 to 79.8dB) pre-surgery and 27.74 dB (± 21.66 dB, range from 1 to 68.75 dB) with CI-use. At T4, the CI-ear had become the better ear for 9 subjects as indicated by a PTA4 difference of more than 10 dB, it remained the worse ear for 2, whereas a difference of < 10 dB was observed for the remaining participants.

### Speech reception thresholds

**Figure 2:**
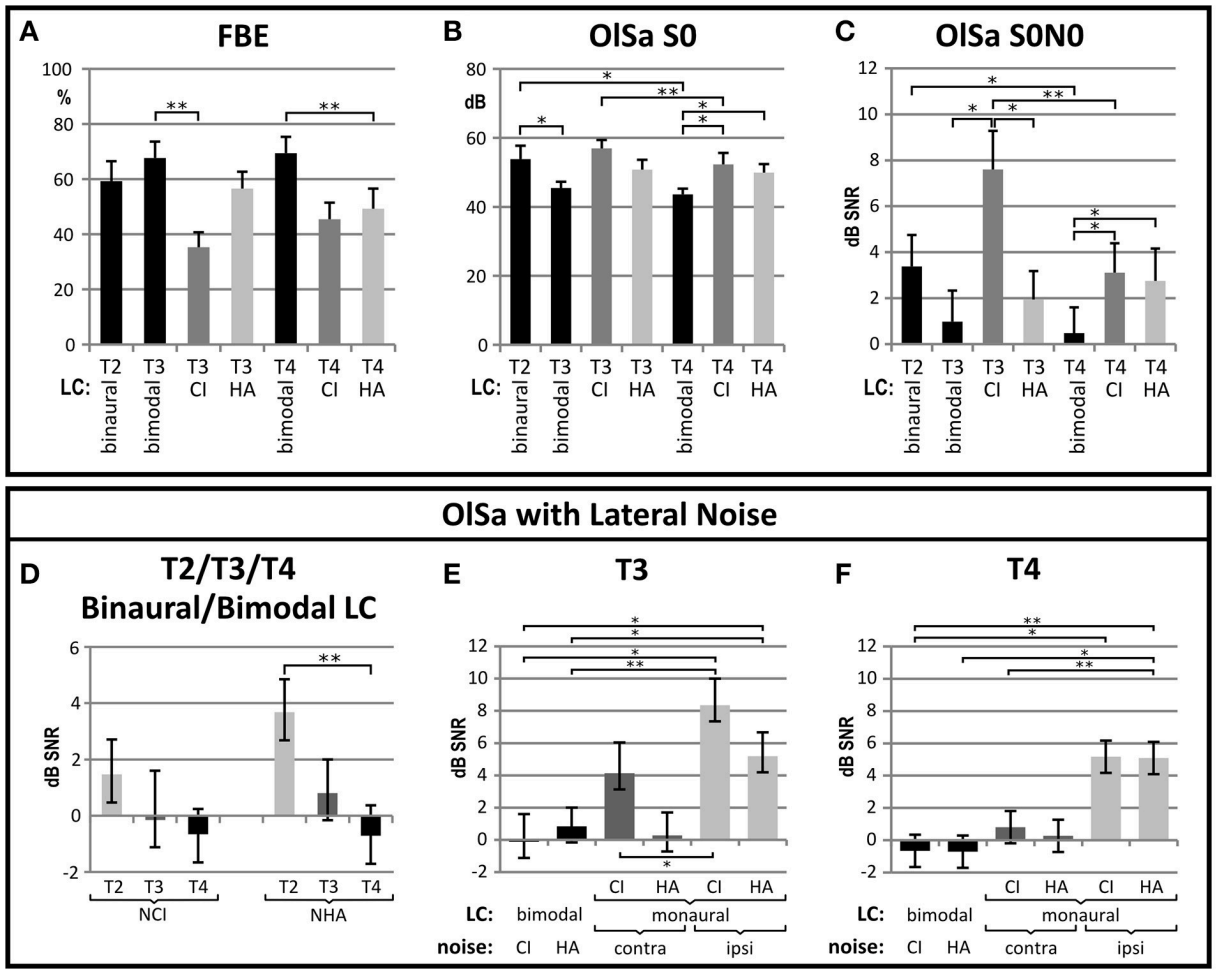
Improvement in speech comprehension between pre- (T2) and post-CI (T3 at 3 months, T4 at 6 months) is shown for **(A)** monosyllables presented in quiet (FBE), **(B)** sentence tests in quiet (OlSa S0), and **(C)** sentence tests presented with noise from the same source (OlSa S0N0) or from the side of the CI (S0NCI) or HA (S0NHA) **(E–F)**. In all graphs, group means are shown with their respective standard error, and significant (*p < 0.05) or highly significant (**p < 0.01) differences with Bonferroni-corrected *post-hoc* comparisons are indicated. **(A–C)** Significant improvements with CI provision are evidenced for the binaural (bin) listening condition (LC), which is bimodal (bim) at T3 and T4. In addition, bimodal LC is often significantly better than monaural LC (CI or HA). **(D)** If noise is presented from the side of the hearing aid (S0NHA) improvement between T2 and T4 is highly significant, while change is insignificant if noise is presented at the CI-side (S0NCI). **(E**,**F)** comparisons of bimodal and each monaural LC are shown for T3 **(E)** and T4 **(F)**. Again LC indicates bimodal or monaural CI- or HA-assisted listening, and noise is presented to the CI or HA-ear for the bimodal LC, and ipsi- or contralateral to the assisted ear for the monaural LC. Performance of the CI ear improves between T3 and T4. At T4 bimodal performance is significantly better if noise is presented at the side of the monaurally assisted ear, but not with noise presented contralateral to that ear.

Group average speech reception thresholds are shown in Figure 2. At T2, speech testing could only be performed in the binaural, best-aided LC, whereas post-operative testing (T3, T4) was performed for the bimodal and both monaural LC.

#### Improvement of binaural SRT and SNR

Binaural speech perception scores in the monosyllable FBE test in quiet improved by an average of 10.15 ± 19.73% (Figure 2). This change did not yield statistical significance (chi^2^ = 2.317; *p* = 0.314). A significant improvement, was evidenced for the OlSa sentence test presented in quiet (S0) with a reduction of 10.24 ± 11.72 dB (*F* = 11.675; *p* = 0.002) in the SRT. When presenting OlSa sentences in competing noise, main effects were highly significant for the S0N0 (reduction of 2.91 ± 4.03 dB; chi^2^ = 12.706; *p* = 0.002) and S0NHA (reduction of 4.99 ± 3.99 dB; chi^2^ = 16.209; *p* < 0.001) conditions. *Post-hoc* comparisons revealed that improvements were significant for the interval between T2 and T4 (Figure 2). If noise was presented at the side of the CI (S0NCI), improvement did not reach statistical significance (reduction of: 2.13 ± 3.87 dB; chi^2^ = 3.851; *p* = 0.146).

#### Improvement of monaural CI-assisted SRT and SNR

Significant improvement of CI-assisted monaural speech comprehension (Figure 2) was observed for OlSa sentences presented in quiet (S0: 7.01 ± 5.72 dB; *z* = 3.516; *p* < 0.001) and for OlSa sentences with noise from the same source (S0N0, reduction of 4.49 ± 4.80 dB; *t* = 3.743, *p* = 0.002), whereas improvements for the other conditions did not reach the Bonferroni-corrected significance level (FBE increase of 10.15 ± 19.75%; *t* = 2.118; *p* = 0.050; S0NCI: reduction of 3.18 ± 5.01 dB; *t* = 2.118; *p* = 0.023; S0NHA: reduction of 3.32 ± 4.55 dB; *t* = 2.917; *p* = 0.011).

#### Bimodal vs. monaural CI-assisted SRT and SNR

At T3, monaural CI-assisted listening was worse than bimodal or HA-assisted listening, but it improved until T4 when bimodal speech comprehension yielded better results than both monaural LC (Figure 2).

At T4, significant main effects were found for FBE (chi^2^ = 8.941; *p* = 0.011; bimodal: 69.41 ± 24.42%; monCI: 45.44 ± 24.80%; monHA: 49.27 ± 30.08%), OlSaS0 (chi^2^ = 7.176; *p* = 0.028; bimodal: 43.60 ± 0.88 dB; monCI: 52.30 ± 13.73 dB; monHA: 49.91 ± 10.22 dB), and OlSaS0N0 (chi^2^ = 9.50; *p* = 0.009; bimodal: 0.48 ± 4.61 dB; monCI: 3.11 ± 5.12 dB; monHA: 2.75 ± 5.79 dB). When noise was presented from the side of the CI (S0NCI) or the HA ear (S0NHA), bimodal speech comprehension did not differ between sides of presentation. Likewise, monaural performance with the CI- and HA-ears did not differ significantly at T4. For noise presented from the side of the aided ear, bimodal SNRs were significantly better compared to each monaural condition (*F* = 16.318; *p* < 0.001; bimodal:−0.68 ± 3.45 dB; monaural CI: 5.17 ± 6.11 dB; monaural HA: 5.09 ± 5.29 dB) while differences did not yield statistical significance with noise presented to the unaided ear (*F* = 0.548; *p* = 0.584; bimodal:−0.68 ± 3.45 dB; monaural CI: 0.81 ± 6.94 dB; monaural HA: 0.27 ± 6.22 dB).

### General health

In addition to bad hearing chronic health conditions were reported by 53% of the participants, and one participant experienced a severe medical incident unrelated to CI-implantation during the study interval. At T2 global health achieved an average of 2.35 ± 0.93 (range from 1 to 4), which is between ok (2) and good (3), and there was not much change until T4 (2.59 ± 1.00 (range from 1 to 4). At T2 and T4 average scores for anxiety and depression (Table 1) remained below the cutoff of 7 for indicating problems in this area, but at both time points expressed a wide distribution (range from 0 to 16) with the highest scores falling into the severe (score 11–14) and most severe (score 11–21) categories. Until T4, values improved marginally for all factors (Table 1), although these changes did not reach statistical significance for either factor. The average GSE-scale score at T2 was 30.82 ± 4.45 (range from 23 to 38) points out of 40.

### Influence of intensive rehabilitation on self-perceived ability and audiometric improvements

Independent of the study, 9 of the 17 study participants enrolled in a 3–5 week long in-patient rehabilitation program at a clinic specialized on CI-rehabilitation, while the other 8 visited regular out-patient CI-rehabilitation services between T3 and T4. This allowed us to compare a group with intensive rehabilitation to a group with regular rehabilitation. Treatment approach in CI-rehabilitation clinics goes beyond auditory training. In short, there are daily single and group auditory training sessions as well as informal conversation groups. Permanent access to auditory training as well as physiotherapy, relaxation exercises, and sports programs are offered. Depending on the needs of the individual patient, tinnitus coaching, balance training, psychological, and social-medical support is offered. Beyond that, interaction with fellow CI-patients is encouraged and the patients do not have to worry about everyday life duties but can concentrate on their hearing. A detailed description of the spectrum typically offered by a CI-rehabilitation clinic can be found in (40).

**Figure 3:**
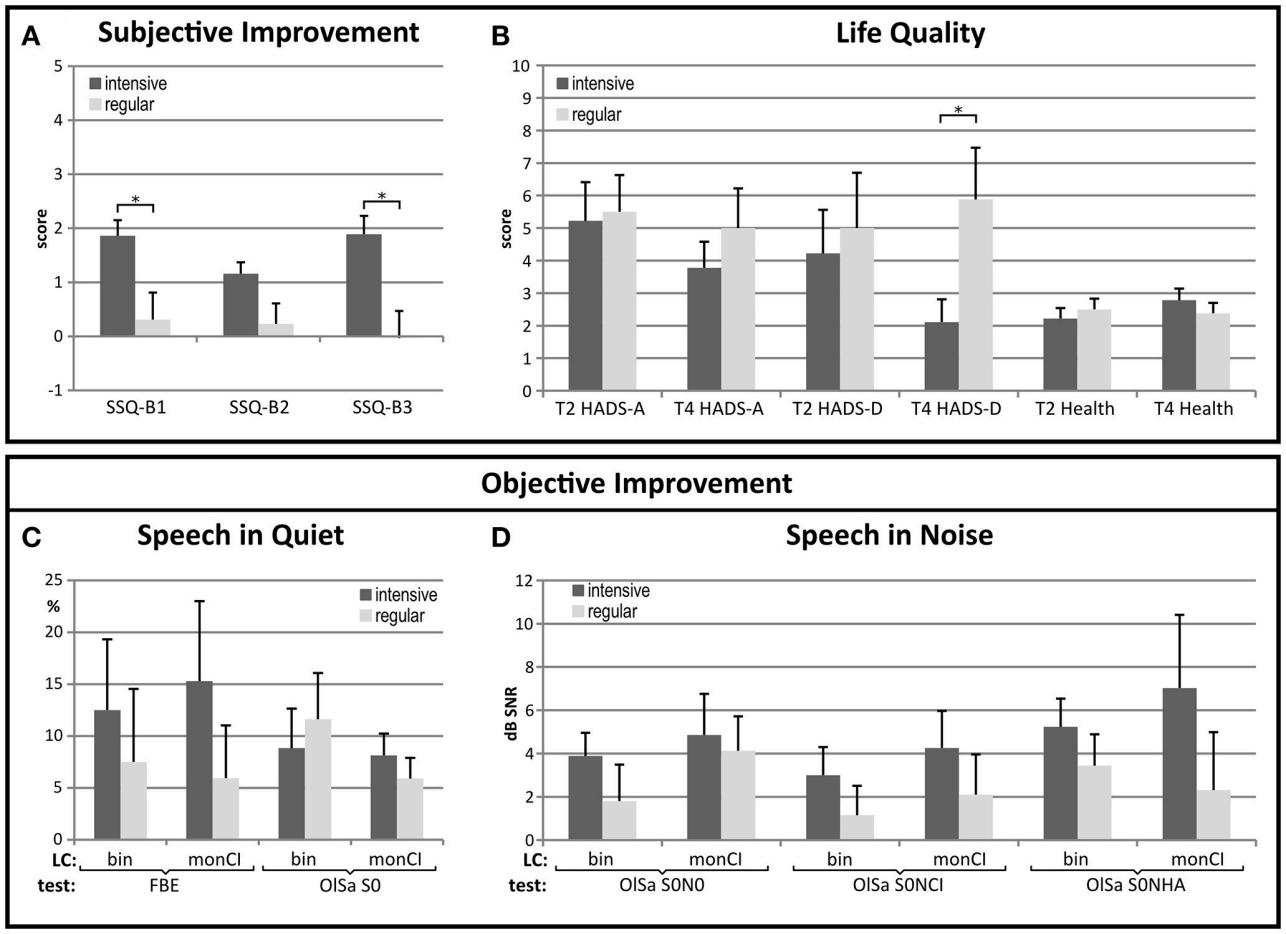
Improvement of self-reported auditory abilities, life quality and audiometric measures in the groups with intensive and regular CI-rehabilitation. **(A)** Subjective improvements for speech comprehension (SSQ-B1) and sound quality (SSQ-B3) were significantly better in the group with intensive CI-rehabilitation. **(B)** The level of depression which did not differ between groups at T2 reached a significantly higher level in the group with regular rehabilitation at T4 (*p < 0.05). Improvements for all but one of the speech comprehension tests **(C**,**D)** were better in the group with intensive rehabilitation, but differences did not attain statistical significance. Bin: binaural, FBE: monosyllable test, HADS-A: anxiety, HADS-D: depression, LC: listening condition, monCI: monaural CI-assisted listening, SSQ-B1-3: scores for self-perceived improvement of speech, spatial and sound quality, T2: pre-surgery, T3: 3 months post-implantation, T4: 6 months post-implantation, statistically significant difference: (*p < 0.05).

At T2, the two groups did not differ regarding PTA4 of the future CI and the HA ears or aided binaural speech comprehension. At the end of the study, improvement of self-reported auditory ability was significantly higher in two of the three SSQ-B scales for the group with intensive rehabilitation according to the Bonferroni-corrected significance criterion of *p* = 0.017 (SSQ-B1: *F* = 7.652; *p* = 0.014; SSQ-B2: *F* = 4.899; *p* = 0.043; SSQ-B3: *F* = 10.355; *p* = 0.006), while improvement in spatial hearing was not significantly better compared to regular rehabilitation (Figure 3).

Improvement of the PTA4 of the CI-ear did not differ between these groups. Also, while initially groups with intensive and regular rehabilitation did not differ regarding levels of anxiety, depression or global health, the level of depression was significantly higher in the group with regular rehabilitation at the end of the study interval (Figure 3). This was due to a decrease in depression for the group with intensive rehabilitation (T2: 4.22 ± 4.02; T4: 2.11 ± 2.09) and a smaller increase in the group with outpatient rehabilitation (T2: 5.0 ± 4.81; T4: 5.88 ± 4.49). Changes between T2 and T4 did not reach statistical significance, however.

### Relation between improvements in self-reported and measured hearing

As scores of SSQ-B1-3 were correlated with *r* > 0.7 and *p* = 0.001 for all 3 comparisons, bivariate correlations were performed between the average of SSQ-B1-3 (SSQ-B_mean_) and the above named factors. In addition, age and duration of hearing impairment did not show a significant correlation with SSQ-B_mean_ (age: *r* = −0.053; *p* = 0.841; duration of hearing impairment of the CI-ear: *r* = 0.192; *p* = 0.461 and HA-ear: *r* = −0.026; *p* = 0.925). As shown in Table 2, correlations of the SSQ-B_mean_ with objective improvements in speech tests in the bimodal LC were low for most comparisons. An *r* > 0.500 was found only for the improvement of monaural CI-aided sentence comprehension in quiet. Noteworthy were the negative correlations with current levels of depression and anxiety and the positive correlation with general self-efficaciousness as assessed by the GSE scale at T2 (Table 2).

**Table 2:** Summary of correlation analyses.

**T4**	**Improvement PTA4-CIear**	**Reduction PTA4 Asymmetry**	**Improvement T2 to T4 binaural Listening Condition**										**Mental Health at T4**		**T2**
			**Speech in Quiet**				**Speech in Noise**						**Anxiety (HADS-A)**	**Depression (HADS-D)**	**GSE**
			**FBE Bin T2/T4**	**FBE Mon CI T3/T4**	**OlSa S0 Bin T2/T4**	**OlSa S0 Mon CI T3/T4**	**OlSa S0N0 Bin T2/T4**	**OlSa S0N0 Mon CI T3/T4**	**OlSa S0NCI Bin T2/T4**	**OlSa S0NCI Mon CI T3/T4**	**OlSa S0NHA Bin T2/T4**	**OlSa S0NHA Mon CI T3/T4**			
SSQ-	0.295	0.020	0.459	−0.337	0.263	**0.646**	0.436	0.477	0.331	0.418	0.258	0.376	−**0.520**	−**0.582**	**0.511**
B_mean_	*0.250*	*0.940*	*0.064*	*0.186*	*0.308*	***0.007***	*0.080*	*0.062*	*0.195*	*0.107*	*0.293*	*0.317*	***0.032***	***0.014***	***0.036***

*Values in upper row represent bivariate Pearson correlation coefficients (r) while values in lower row indicate p-levels (italic). Variables in bold with an r > 0.500 and a p-level < 0.05 were included in the regression analysis. The Bonferroni-corrected significance level was 0.0028*.

#### Regression analyses

The three factors with *r* > 0.500 and *p* < 0.05 in the bivariate comparisons (Table 2) were included in the forward and stepwise regression analyses that were performed separately with the SSQ-B1-3 scales to determine the most appropriate model to account for subjectively perceived bimodal benefit. The coefficient of determination (*R*^2^) is a measure related to the proportion of variance that is explained by the independent variables. *R*^2^ above 0.5 are considered to describe a significant portion of the variance indicating a valid model. Explained variance was highest for spatial hearing, with current level of anxiety being the major predictor for self-perceived improvement on this scale. Predictions were less accurate for SSQ-B1 and SSQ-B2, with the major predictor being general self-efficaciousness at T2 for subjective improvement in speech comprehension, while improvement of sentence comprehension in quiet for monaural CI-assisted listening between T3 and T4 was the main predictor for self-perceived improvement in sound quality (Table 3).

**Table 3:** Results of regression analyses.

**Predictors**	**Dependent variable**	***R*^2^ and Significance of the model**	**Significant predictors**
- Improvement of monaural CI-aided OlsaS0 between T3 and T4	SSQ-B1 speech comprehension	Significant *R*^2^ = 0.587; *F* = 3.902; *p* = *0.033*	GSE at T2 B = 0.611; *F* = 8.328; *p* = *0.012*
- Depression at T4	SSQ-B2 spatial hearing	Significant *R*^2^ = 0.615; *F* = 4.386; *p* = *0.023*	Anxiety at T4 B = −0.647; *F* = 10.070; *p* = *0.007*
- Anxiety at T4	SSQ-B3 quality of sound	Significant *R*^2^ = 0.553; *F* = 3.408; *p* = *0.048*	Improvement of monaural CI-aided OlsaS0 between T3 and T4 B = 0.582;
- GSE at T2			*F* = 7.155; *p* = *0.018*

*Separate multiple regression analysis were performed for each of the SSQ-B scales with all the predictors listed in the left column. Significant predictors for the SSQ-B scales differ as shown in the right column. The coefficient of determination (R^*2*^) is a measure related to the proportion of variance that is explained by the independent variables. R^*2*^ above 0.5 are considered to describe a significant portion of the variance indicating a valid model. GSE, General Efficaciousness Scale (38). p values in italics*.

## Discussion

Objective of this study was to examine self-reported improvements of auditory ability during transition from HA-assisted to bimodal hearing, to relate these to audiometric improvements evidenced by pure tone and speech audiometry, and to explore which factors besides hearing influence self-reported auditory ability. An advantage of our study is the uniform type of CI-provision and that all participants used a HA on the contralateral ear. Novel findings from the study are (1) that self-perceived improvement in speech comprehension depends on general self-efficaciousness, while (2) self-perceived improvement in spatial hearing is negatively influenced by current level of anxiety, and (3) that intensive CI-rehabilitation has a positive influence on self-perceived improvement in auditory abilities and on the level of depression.

Self-reported improvements in auditory ability as assessed by the SSQ-B were significant but small and the correlation between self-perceived and audiometric improvements was low. This is in general agreement with the recent literature (1, 5, 20, 30, 41). Reasons for this finding will be discussed.

Only monaural CI-assisted improvement for sentence comprehension in quiet proved to be a significant predictor for self-reported improvement, namely for the improvement in sound quality. As everyday listening situations are often impeded by noise, this was unexpected and may have been due to the type of noise that was used. Alternatively speech comprehension in quiet may be the most common situation CI-users encounter in their daily life. This appears feasible as many of our study participants were older and most common communication situations appear to be in quiet for the older age groups (13). In addition, as difficulty with intelligibility is most pronounced in noisy surroundings, the hearing impaired may avoid these situations. This finding is in accordance with the results of a previous study (27) which also found the highest correlations between patient-reported benefit, here assessed with the Nijmegen Cochlear Implant Questionnaire, and sentence comprehension in quiet. Further, it suggests that the common clinical practice of assessing CI-aided speech comprehension in quiet is a valid approach.

### Improvement of self-reported auditory ability

Self-report questionnaires are an economic means for assessing the perceived real-world benefit of aided hearing and allow a more comprehensive insight into the remaining handicap than speech comprehension tests in the laboratory (18). As measure for patient-reported improvements we used the benefit version of the SSQ, the SSQ-B, which allows subjects to directly rate the benefit they perceive with their new hearing provision (21, 22). Moreover, SSQ and likewise SSQ-B focus on auditory abilities as opposed to handicap and they address spatial hearing in a separate subscale. Although significant, subjective improvements are small particularly given the substantial improvements in CI-assisted listening over the study interval. This implies that the questions posed by the SSQ/SSQ-B address aspects of hearing that are not effectively measured with current audiometric assessments. As observed by Noble and Gatehouse (19) when investigating the effects of uni- vs. bilateral HA fitting after 6 months of use, binaural benefit exists for dynamic spatial hearing regarding distance and movement of auditory objects, for rapidly switching and divided attention, and regarding listening effort, but not for tasks like speech comprehension in quiet or in static noise. Furthermore, in the real world behavioral adaptations like lip-reading or strategic positioning serve to improve comprehension problems (42). Therefore self-perceived binaural improvement may be lower than improvement observed in situations that solely rely on auditory sensitivity, for instance during audiometry.

### Patient expectations

Patient expectations are twofold. One is the expectation that CI-provision improves hearing ability. This point was addressed at T2 by a question asking participants what they expected from their CI. Our study participants expressed global expectations for hearing improvement and hoped for better participation in social situations. A related factor is patient expectations regarding time course and the result of CI-rehabilitation, which was suggested to play a significant role in self-perceived improvement of auditory communication (43). With various medical conditions, patient expectation was found to be a powerful predictor for future well-being and thus related to success of medical treatments, and a growing body of research indicates that addressing and optimizing patients' expectations can be an important contribution to medical treatment (44).

The group that enrolled in intensive rehabilitation experienced significantly higher subjective improvements than the group with regular rehabilitation. It is possible that, although unvoiced, improvements were expected to happen with little effort from their side in the group with regular rehabilitation resulting in unmet expectations associated with worse scoring. In contrast, those applying for intensive rehabilitation actively pursued their training experience and had more contact with experienced CI-patients, which may have reduced expectation to a more realistic level associated with better scoring as suggested by a previous report (43). Taken together realistic expectations appear to be important for perceived benefit and should be explored in more detail.

Another expectation is that demands resulting from CI-provision can be mastered. This aspect was addressed at T2 with the General Self-Efficaciousness Scale (GSE) by Schwarzer and Jerusalem (38). General self-efficaciousness as originally defined by Bandura (45) is based on the assurance to master new and challenging tasks successfully, relying on one's own abilities. Present average pre-surgery GSE score is comparable to that found for various clinical and non-clinical samples (38, 39, 46), and it is the most important predictor for self-reported improvement of speech comprehension. Across different patient groups, high scores in the GSE scale are related to more frequent use of active problem-focused coping as opposed to passive coping (39). In a study on the association between hearing status and psychosocial health with data collected in combination with the Dutch National Hearing Test (15), GSE increased with decreasing hearing in HA users in their forties. This was seen as an indication of compensatory behavior. Among patients with cardiovascular diseases higher GSE-scores are related to a stronger intention to exercise and to the expectation of positive outcomes, while cancer patients with higher general self-efficaciousness are less depressed and report better health and life quality (39). In summary patients with higher general self-efficaciousness tend to follow medical advice more closely and are more content with the outcome.

### Mental well-being

Current level of anxiety was the dominating predictor for subjectively perceived improvement in spatial hearing. In addition, the level of depression, which did not differ between groups with intensive and regular rehabilitation at T2, was significantly lower in the group with intensive rehabilitation at T4. This was associated with significantly higher self-reported improvements in speech comprehension and sound quality in this group. As in other studies (47, 48), levels of anxiety and depression were significantly correlated in the present study (T4: *r* = 0.586; *p* = 0.013). Interestingly, Noble et al. (29) found that bimodal provision is associated with significantly greater post-implant emotional distress than bilateral CI, and this could not be accounted for by pre-implant distress. In a sample of elderly hearing aid users anxiety was seen to correlate significantly with self-perceived auditory disability and handicap but not with PTA (49). Epidemiologic studies evidence significant correlations between depression and hearing status and even more so with self-reported functional hearing (50), while mental health in general is found to correlate significantly with hearing in younger and older age groups (16). Also, in a 30 year follow-up report on otosclerosis patients with stapedectomy and some of them using hearing aids, mental well-being as assessed by the SF36 questionnaire correlated significantly with all three scales of the SSQ but not with hearing sensitivity (51).

The significant contribution of anxiety to self-perceived spatial hearing may be due to the circumstance that bimodal patients with increased levels of anxiety avoid exposure to situations which require verbal communication and orientation in space, which in turn limits training and ultimately leads to poorer improvements in spatial hearing. Based on the present data it cannot be determined, whether study participants with a higher level of anxiety avoid exposure or whether they do not acclimatize despite ample exposure. Therefore influence of anxiety on spatial hearing ability could also be an indication of altered neural plasticity which has been observed during states of enhanced anxiety and depression (52–54). Neuronal plasticity is necessary to develop fusion of the electrically and the acoustically transmitted signals in the auditory brain, which should be particularly important for the development of bimodal spatial hearing as assessed by the SSQ-B2.

### CI-rehabilitation

Auditory training is an important aspect of CI rehabilitation, in particular during the first months after implantation. Current trends promote home-based auditory training for the rehabilitation of CI-patients assuming that this is sufficient to achieve optimal performance in daily life (55). In contrast, our results suggest that increased intensity of therapeutic intervention addressing psychosocial factors and interaction with fellow CI-patients may be of importance for the rehabilitation process. Data by Tang et al. (56) do also support the value of post-operative rehabilitation in enhancing quality of life gains associated with CI and suggest that reduction of psychosocial barriers may result in gains of communication efficacy and within other functional domains. Another important aspect in this context appears to be extended contact with professionals as well as CI-patients. Therefore intensive inpatient rehabilitation programs may address non-auditory problems more effectively and thereby lead to improved self-perceived benefits and potentially also to accelerated improvements in speech comprehension tests as suggested by a previous study (40). Further elucidation of benefit associated with different types of CI-rehabilitation is warranted.

### Limitations

As performance on speech tests depends on the type of speech and noise material, other test configurations may have yielded deviating results. Furthermore, the study size was small as usual for prospective studies with CI-patients, and data show considerable across-subject variability. Also, the time interval between switch-on of the CI and the final evaluation comprised an average of 6 months. This may have been too short to have gained enough individual experience with bimodal listening in all situations that are assessed by the SSQ-B (57). Other longitudinal studies with CI-patients evidence however, that most improvement seen in the SSQ takes place during the first 6 months and that a stable plateau is reached within this interval (7). Also, improvements in sentence comprehension in quiet and noise essentially reach a stable plateau within 6 months of CI use, while improvements in the FBE test continue until 12 months post-implantation (58). An additional factor that has to be taken into account in longitudinal studies on bimodal hearing is the possibility that hearing in the non-implanted ear may worsen. This was evident for some of our subjects. Therefore conclusions have to be interpreted with caution and await reproduction in future studies.

## Conclusion

Bimodal provision was well accepted, as indicated by joint use of CI and HA during most of the day. Data suggest, however, that improved audiometric results do not necessarily coincide with a reduction in auditory communication difficulty. Thus the SSQ-B provides useful information regarding problems with auditory communication and possibly also with psychological factors related to auditory ability, but it does not qualify as a sole measure for the improvement of auditory abilities as results are influenced by non-auditory factors. Divergent developments in the groups with intensive and regular CI rehabilitation indicate that self-perceived auditory improvements following CI-provision can be positively influenced. Therefore, expectations and motivation of future CI-patients should be characterized in more detail, and mental health needs to be monitored and eventually treated in the course of CI-rehabilitation. This may help to identify subgroups of patients who would benefit from certain types of intervention during CI-rehabilitation.

## Author contributions

EW-F designed the study, collected and analyzed the data, and wrote the manuscript. TB data collection and critical review. SH data analysis. NR critical review. JS recruitment and critical review.

### Conflict of interest statement

The authors declare the following interests. This study was partly funded by Advanced Bionics AG, Staefa. Switzerland. Advanced Bionics AG manufactures the device under investigation in this study. This does not alter the authors' adherence to all the Frontier policies as detailed online in the guide for authors.
